# Designing Laboratory for IoT Communication Infrastructure Environment for Remote Maritime Surveillance in Equatorial Areas Based on the Gulf of Guinea Field Experiences

**DOI:** 10.3390/s20051349

**Published:** 2020-02-29

**Authors:** Ranko Petrovic, Dejan Simic, Dejan Drajic, Zoran Cica, Dejan Nikolic, Miroslav Peric

**Affiliations:** 1 Faculty of Organizational Sciences, University of Belgrade, Jove Ilica 154, 11000 Belgrade, Serbia; dejan.simic@fon.bg.ac.rs; 2 Vlatacom Institute of High Technologies, Milutina Milankovica 5, 11070 Belgrade, Serbia; dejan.nikolic@vlatacom.com (D.N.); miroslav.peric@vlatacom.com (M.P.); 3 School of Electrical Engineering, University of Belgrade, Bul. Kralja Aleksandara 73, 11120 Belgrade, Serbia; ddrajic@etf.bg.ac.rs (D.D.); zoran.cica@etf.bg.ac.rs (Z.C.)

**Keywords:** internet of things, satellite communication networks, laboratory environment, maritime surveillance, marine systems

## Abstract

The steady increase of the world population and economy leads to an increase in both types and amounts of goods transported over seas, which further inevitably leads to an increase of criminal activities in the maritime arena. In order to stifle criminal activities nations are forced to develop sophisticated sensor networks. The backbone of any sensor network is a communication network which connects all sensors with the command centers, most often located hundreds of kilometers away from the sensors. In developing countries, communication networks are very often poorly developed, leaving only satellite links as somewhat reliable means of communication. Henceforth, in this paper, a laboratory for the Internet of Things (IoT) communication infrastructure environment designed to facilitate maritime sensor network design process in areas where communication network is dependent on data transfer over satellite links is presented. In order to successfully describe and develop a laboratory for IoT communication infrastructure environment, necessary data are collected during the design and deployment of a maritime surveillance network in the Gulf of Guinea. The main advantage of the proposed laboratory environment is the inclusion of satellite link simulation in the IoT laboratory environment. This feature provides an opportunity to cover a much broader scope of IoT solutions compared to other IoT laboratories.

## 1. Introduction

During the last few decades, organized crime in the maritime arena is practically flourishing, threatening both the secure flow of the goods from the Exclusive Economic Zones (EEZ) [1] and Territorial Sea [2] alike. Sometimes, even the lives of the participants in marine traffic are at danger, forcing the actions of international organizations such as United Nations (UN) [3] and/or European Union (EU) [4], since nations which have jurisdiction over threatened waters have very limited resources. Apart from countries and international organizations, there are other participants in the marine traffic which are directly hit by illegal activities in the maritime arena. For example, according to the World Wildlife Fund, fishing companies are losing billions of dollars every year due to illegal fishing [5]. More importantly, overly excessive fishing can lead to the complete depletion of biological resources and thus endanger the marine ecosystem to the point of no return [6]. On the other hand, according to [7], maritime piracy is becoming quite common in some regions of the world. Attacks are mostly performed in order to hijack a vessel and request a ransom for crew and vessel itself or to steal valuable cargo such as crude oil. Regardless of the final goal of the pirate attack, it is clear pirate attacks must be stopped. The very same is true for illegal fishing, which is probably even more important, since permanently damaging oceans ecosystems will definitely endanger a human survival on this planet.

In order to provide a safer maritime environment, firstly a sensor network for continuous monitoring of maritime activities in territorial sea and EEZ must be established. On the other hand, since EEZs are huge bodies of water that can cover hundreds of thousands of square kilometers, complete monitoring is much easier said than done. To the best of our knowledge, there are only two ways to achieve complete EEZ monitoring. The first approach utilizes optical and microwave sensors on platforms such as satellites and airplanes, thus avoiding the limitations of the sensors, but this introduces limitations in the platform. The most limiting factor is the interrupted data availability, since no airplane is able to stay in the air constantly during the whole year and during all weather conditions. Meanwhile, satellites, which are orbiting around the Earth, are over the zone of interest for a limited time only.

The other approach uses a network of High-Frequency Surface Wave Radars (HFSWR) [8,9] to ensure the constant surveillance well beyond the horizon. Since the price of the HFSWR radar network is significantly lower than the combined cost of the aforementioned sensors and platforms in the first approach, and due to the fact that those sensors provide limited data available throughout the whole year, it is clear why HFSWR networks are gradually becoming the sensors of choice for maritime surveillance at OTH (Over-the-horizon) distances. The HFSWRs which are used as centerpieces of maritime surveillance networks are described in detail in [10]. Besides HFSWR which is crucial for large vessel detection and tracking at OTH distances, maritime surveillance systems are equipped by multi-sensor electro-optical devices (MEOD) and conventional microwave radars (MWR). Microwave radars require line-of-sight conditions between radar and target for its operation so its range is limited by the height of their installation. In order to provide surveillance during day and night time, and in various meteorological conditions as well, MEOD combines various spectral ranges like visible light (color) camera and thermal cameras. Due to naval condition for thermal camera medium wave infrared (MWIR) spectral band is used which gives ship detection ranges typically up to 20 km [11]. Additionally, MEODs are equipped with a long-range laser range finder (LRF), which precisely determines distance even to very small vessels, no matter of its material [12]. For providing good situation observation, data fusion from various sensors are necessary. There are three scenarios for accomplishing this task: site-level fusion, command and control center fusion (C2) and combined fusion. Thus, networking between sensors and fusion elements is a very important component of remote maritime surveillance system implementation whose effects should be analyzed in detail both in the system concept phase, early system implementation phase in laboratories and after deployment fine system tuning phase.

The crucial component in both approaches is a communication layer between the sensors, command and control centers (C2 centers) and participants in marine traffic. This component is easily provided in developed countries, but in developing countries, it is most often not the case. To make an already bad situation even worse, the majority of illegal activities are done in waters belonging to developing countries, where even an electrical power network is lacking, not to mention communication infrastructure. Luckily, due to available satellite networks [13,14,15], IoT-based [16,17] communication infrastructure is available in nearly all corners of the world and can be deployed in order to provide communication infrastructure for maritime sensor networks. Using this approach, data collected from sensors in the field can be available not only to the parties in the monitored area, yet data can be delivered to all those who have interest in them, anywhere in the world. In other words, organizations situated in Europe or North America can monitor the situation in affected areas in real-time, allowing them to directly guide and synchronize the course of the action in the field located tens of thousands of miles away.

In this paper, experiences gained during the building of sensor network for coastal and OTH monitoring in the Gulf of Guinea based on IoT satellite communication infrastructure, are presented. The communication network is primarily used to connect all sensors dispersed over 800 km of coastline with command centers located inland, thus allowing real-time monitoring of hundreds of thousands of square kilometers of the sea. Secondly, this very same IoT based approach allows monitoring of the very same maritime situation in real-time from a location in Europe located more than 5000 km from the sensor network. Based on these experiences, we propose a laboratory for IoT communication infrastructure environment that can help in building similar sensor networks in other locations of interest as well. The proposed environment is based on free and open-source tools, namely WANem [18,19], GNS3 [20], Strong Swan [21] and Quagga Routing Suite [22] which make it an attractive solution for network designers during the network planning process. Our experiences show that the critical point in communication infrastructure is the satellite link. Satellites provide great communication coverage, however, the provided link bandwidth is limited and propagation delay is significant. The proposed laboratory for IoT communication infrastructure environment allows testing different values of network parameters, including the critical satellite link parameters, in order to help network designers meet desired communication infrastructure behavior and performance. Tools used for testing real network and laboratory IoT communication infrastructure environments are also open-source tools, namely Wireshark [23], Zabbix [24] and Iperf [25].

Since IoT is a very attractive topic that attracts a lot of attention, various IoT laboratory environments are already proposed in the literature. Most of the proposed laboratories are located on campuses and mainly serve for educational purposes [26,27,28]. In [27] the author presents a wisdom laboratory based on IoT for campus purposes, such as student education or improvement of the quality of life and study. The main goal of such laboratories is to introduce students to IoT and give them basic knowledge in the IoT field. These IoT laboratories for education purposes are typically equipped with common sensors like temperature and humidity sensors, and controllers like Arduino and Raspberry Pi [26]. In [26], the authors have designed and implemented a smart laboratory system using IoT technology for remote energy monitoring and to control appliances inside their lab. By employing the proposed system, the total energy consumption can be reduced in the observed campus. Students learn to program and design IoT applications and to remotely control and monitor IoT sensors and devices. The main focus of these laboratories is on the smart home, smart city and other similar IoT topics where special attention is given to improving energy efficiency and improving quality of life aspects [26,29]. In the scope of the VICINITY project, a platform linking various ecosystems providing “interoperability as a service” for infrastructures in the IoT was built and demonstrated. The following use cases are analyzed: Intelligent Building System, Smart Parking, Smart Energy System and eHealth and Assistive Living Home [29]. The communication part of these laboratories mainly comprises common communication technologies and standards like Ethernet, ZigBee, Bluetooth. Besides education purposes, IoT laboratories are also typically used for efficient IoT application development and testing [29]. Usually, IoT laboratories are concerned with pure IoT aspects like IoT applications and IoT devices, and not with the communication infrastructure and communication link parameters that can have a serious impact on the overall IoT ecosystem. In [30], a model to evaluate latency, jitter and control protocols on IoT applications with geographically distributed elements is proposed. A model to correct measure deviations is also presented. Again, the most common usage of these laboratories is for smart home and smart city solutions [29].

The laboratory environment proposed in this paper focuses mainly on the IoT communication infrastructure and its impact on a maritime surveillance network, unlike the other laboratories. The laboratory was extensively used during the design process of multi-sensor data integration algorithms [31,32] in order to provide the designers of the aforementioned algorithms insight into real communication infrastructure behavior. In this way, the algorithms are mostly tuned to the real working environment in the laboratory environment and later integrated into the real maritime surveillance network, thus minimizing the impact of the tuning process on the operational readiness of the maritime surveillance network. The proposed laboratory environment development was an iterative process during the design of the maritime surveillance network in the Gulf of Guinea since the data acquired from the real network were used as feedback to optimize the parameters of the laboratory environment. The usage of the proposed laboratory environment in the design of the real maritime surveillance network in the Gulf of Guinea validates that the proposed laboratory environment is useful in the overall maritime surveillance network design.

Special attention is also given to the impact of a satellite link on the overall IoT network which is not usually covered by other IoT laboratories as they target other IoT solutions where satellite link is not used, thus extending IoT solutions coverage compared to already available IoT laboratories. Inclusion of the satellite link impact on the overall IoT network performance gives the possibility to network designers to plan and implement IoT networks that includes satellite communication as an essential part of the communication infrastructure. As stated earlier in this introduction, maritime surveillance is very often implemented in countries with poor communication infrastructure and using satellite communication is a necessity. Since most of the IoT networks do not use satellite communication, usually IoT laboratories do not include satellite links or simulation of the satellite links. This inclusion of the satellite link simulation in the proposed laboratory environment extends the usage of the proposed laboratory to a much larger set of IoT applications. Satellite link simulation allows network designers to reduce expensive satellite link rent costs during the testing phases in the design process and to reduce the negative impact on user traffic during the production phases in the design process.

The rest of the paper is organized as follows: in Section 2 short description of the deployed Sensor Network for remote maritime surveillance and its communication infrastructure is given. Section 3 deals with the determination of existing communication infrastructure parameters, while Section 4 provides requirements for the proposed laboratory for IoT communication infrastructure environment and its realization. Usage of the proposed laboratory environment during the design and testing of multi-sensor data integration algorithms for vessel tracking at over the horizon distances is presented in Section 5. Potential usage extension of our proposed laboratory environment to other IoT based applications besides maritime surveillance is also presented in Section 5. Finally, Section 6 provides conclusions and directions of future work.

## 2. The Deployed Sensor Network and Its Communication Infrastructure

**Figure 1:**
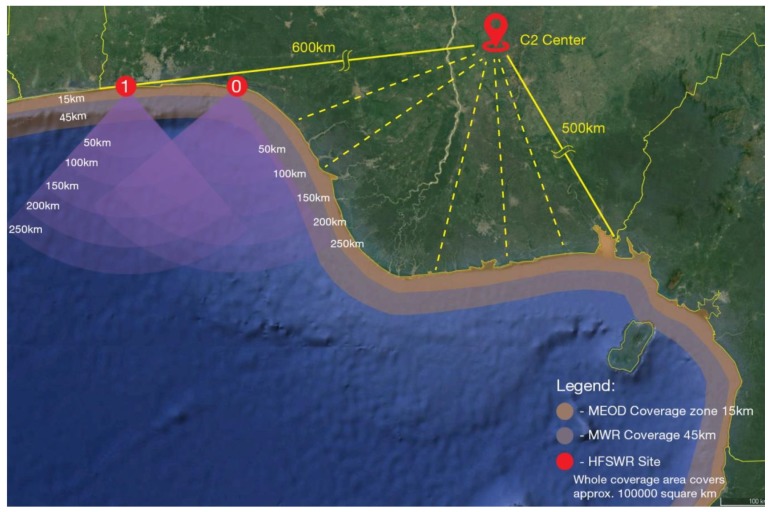
Deployed sensor network coverage area.

The Sensor Network used in this experiment is deployed in the Western part of the Gulf of Guinea and it consists of:

- Two HFSWRs with a nominal range of 80 nautical miles (approx. 150 km) for Bonn express class of vessel [33] during night-time and sea states [34] up to 3. For larger vessels and during the day-time, the range can extend even beyond 125 nautical miles (approx. 230 km). Regardless of the time of day and vessel size, angle coverage is set to 120 degrees. HFSWR network coverage area currently covers the western part of the Gulf of Guinea as shown in Figure 1,
- Twelve coastal sites equipped with microwave (X band) radar systems (MWRs), multi-sensor electro–optical devices (MEODs) [11], Laser Range Finders (LRFs) [12] and Automatic Identification System (AIS) receivers,
- There are also data provided from satellite AIS provider, but since the data are delivered directly to the main command and control center (i.e., that data are not transferred from remote locations) it will not be further examined here.

The coverage area of the deployed maritime surveillance network is shown in Figure 1.

The rest of the aforementioned sensors is deployed on coastal sites situated in the way to cover blind zones of the HFSWRs and to provide monitoring of sea areas near the shore. Please note that, at this moment, only site 0, shown in Figure 1, is at the same time equipped with HFSWR and Coastal sensors. Overall, sensors are dispersed over 800 km of the shoreline and connected to 3 regional control centers, which are then connected to the main control center located more than 400 km inland. Please note that we are not in liberty to disclose exact locations of the sensor locations nor any additional information regarding the used sensors due to Non-Disclosure Agreement (NDA) in this project, but we have briefly illustrated zones covered by MEOD and MWR.

Since a good quality of service is mandatory in this kind of applications, it is necessary to define possible problems through describing sensors data rate requirements:

- Microwave radars and HFSWRs require a small amount of data, but all data need to be delivered in real-time,
- MEODs require a large amount of data in real-time,
- Although LRFs require a relatively small amount of data for measurement results, for measurement triggering accurate video image transmission with low latency is required. Thus, remote usage of LRF is the most demanding service regarding transmission network performance requirements in this network.

**Figure 2:**
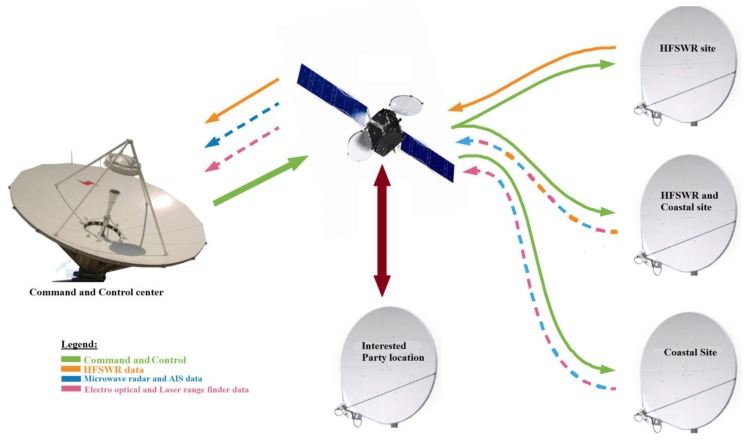
Communication infrastructure between the command and control center and remote locations.

Since, practically, worldwide availability of data is needed, the remote locations and command and control center (located deep inland) are connected over the satellite links. The general layout of the communication channel from the remote site to the command and control center is shown in Figure 2. It is clear from the communication infrastructure shown in Figure 2 that satellite communication plays a central role in connecting all infrastructure components. Therefore, special attention has to be given to the analysis of the satellite links during the communication infrastructure planning in order to avoid serious failures and malfunctions of the overall system later.

## 3. Determination of Existing Communication Infrastructure Parameters

### 3.1. Network Parameters

Each network system consists of a set of network elements, such as routers, switches, firewalls, links and applications, where each of these elements in its own way affects the characteristics of the network. The state of a network system is a set of relevant variables and parameters such as bandwidth, throughput, latency, jitter, packet loss, packet corruption, the random disconnection that describes the system in current conditions [35,36,37].

The bandwidth of a satellite link is a theoretical maximum amount of data that could be sent over that link, while the throughput is an amount of data that actually and successfully travel through the satellite link. Bandwidth and throughput are typically measured in bits per second [35,36,37].

Propagation delay is the time required for packet to propagate from one end of the link to another. Ping is a popular networking application used to test connectivity to a remote location, whether a particular host is up and reachable. Ping effectively measures latency, the round-trip-time (RTT), which includes all delays on the path, as a result of packet propagation through the network, packet processing in various network devices, etc. [35,36].

A parameter that may be even more important in real-time applications than the delay itself is jitter, which represents variations in delay from packet to packet. This parameter is especially important for real-time applications such as Voice over IP (VoIP) and video surveillance.

Since the communication network is practically a packet network, packet losses and bit error rate are also important parameters that reflect the link condition quality. For example, if the deep fading conditions are frequent on the wireless link, then more bits will be received incorrectly, and as a consequence more packets will be dropped, i.e., lost. Bit error rate (BER) is defined as the number of corrupted bits in relation to the total number of transmitted bits. Packet error rate (PER) that reflects the packet losses is defined as the number of lost packets in relation to the total number of transmitted packets. These two parameters are more important for wireless links (like satellite links), as typically the wired links are reliable, i.e., wired links have very low values of PER and BER [35,36].

Unexpected network outages that include the loss of network resources due to device malfunction, power failure, misconfiguration of equipment, but also due to natural disasters such as floods, earthquakes, etc., represent a particular problem in network normal operating conditions [35,36].

Given Figure 2, it is clear that satellite communication is the heart of the communication infrastructure. Therefore, the possibility of simulating a particular satellite link is of exceptional importance for analyzing its performance, its impact on the overall communication and its implementation in practice. Our proposed open source laboratory for IoT communication infrastructure environment gives a possibility to test the influence of satellite links on overall communication performance. The proposed laboratory environment allows us to simulate satellite link parameters (propagation delay, bandwidth, PER, BER, jitter and packet loss) and test their impact on the overall communication infrastructure performance [35,36].

Table 1 shows network parameter requirements for different sensor types. One of the goals of the proposed laboratory for IoT communication infrastructure environment is to test IoT sensors behavior in various network conditions, whether the given requirements are fulfilled or not.

### 3.2. Estimation of Satellite Link Parameters

The satellite network is a complex system that delivers and provides many significant services such as Internet access, communications, navigation, broadcasting, tracking, etc. As stated before, the satellite network is a critical component of the communication infrastructure shown in Figure 2. Therefore, it is important to simulate the satellite link parameters properly with as high as possible accuracy. Simulation can provide an insight into desired satellite link conditions. However, in order to bring simulation results closer to the realistic conditions, the satellite link conditions for particular locations should be measured and estimated. In this way, the simulation can help the network designers to plan the optimal combination of the communication infrastructure and user services that run on that communication infrastructure.

**Figure 3:**
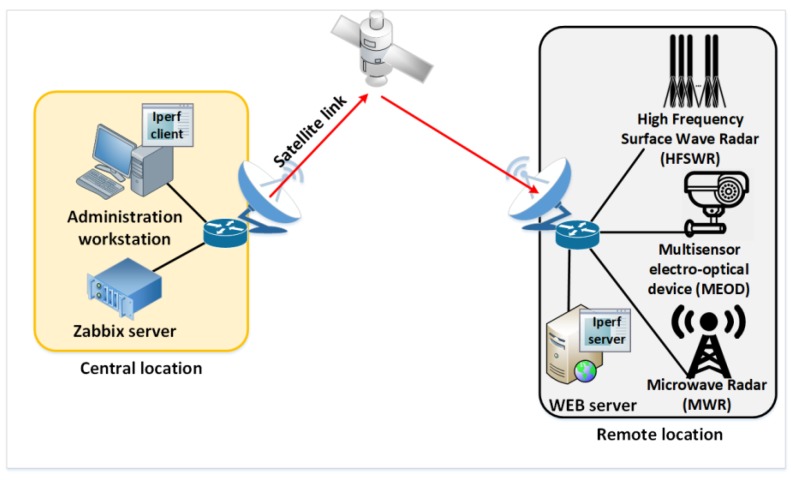
Network parameters measurement of the real satellite link.

In order to accurately simulate a particular satellite link, it is necessary to collect as much information as possible about its characteristics as well as the type of traffic that is transmitted. Information such as available bandwidth, delay, jitter, packet loss, traffic type, network protocol, transport protocol, frame rate, frame size, etc., can be obtained using various software tools for network analysis (Wireshark [23], Zabbix [24], iPerf3 [25]). The system setting for collecting the mentioned parameters is shown in Figure 3.

Zabbix [24] is an enterprise open source monitoring software for networks and applications. It is designed to monitor and track the status of various network services, servers, and other network hardware. Zabbix server is located on the central site and it collects data from all devices that are on remote locations (routers, switches, electro-optical devices). Based on yearly data collection (each month few network parameters measurements are performed in order to avoid traffic overload in the network), it is found that the average value of ICMP ping to the router on a remote location is 661.88 ms. The average ICMP loss for the same period of time is 1.43%. The average value of jitter determined with open-source tool that combines the functionality of the traceroute and ping programs in a single network diagnostic tool is 31.9 ms.

The results of conducted measurements for the observed satellite link using Iperf3 are shown in Table 2.

The goal of the satellite link parameter measurements is to achieve a realistic satellite link model in the laboratory for IoT communication infrastructure environment. In order to achieve as high as a possible accurate estimation of the satellite link without causing regular traffic disturbance, 6 independent measurements were conducted each day over the course of 12 months, i.e., one measurement was conducted every 4 h each day. Each measurement comprises 4 traffic scenarios, three TCP scenarios which differ in number of parallel streams and UDP scenario. Each traffic scenario in measurement lasted 60 s in order to avoid excessive regular traffic disturbance. Table 2 shows some typical measurement results, where the first row represents the worst case, and the others rows represent the most frequent measurement results. The worst cases are typically caused by poor weather conditions such as heavy rains that deteriorate satellite link conditions. The UDP does not have an error control, thus in poor link conditions, many packets are lost and achieved throughput is decreased. The number enlisted beside TCP regards the number of parallel TCP streams. As Table 2 shows, TCP achieved throughput is stable. For example, the achieved throughput in the case of 10 parallel streams is roughly 10 times greater than achieved throughput in case of 1 parallel stream.

**Figure 4:**
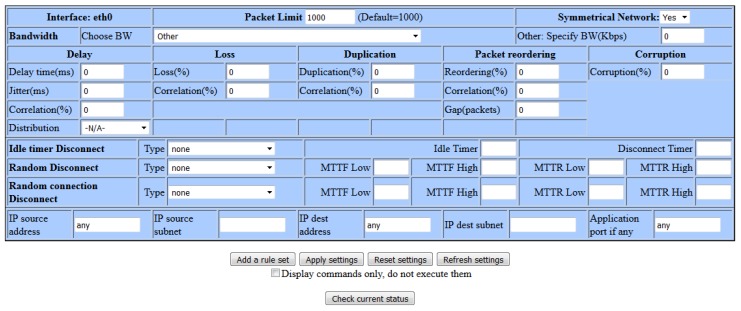
WANem emulator.

After collecting the appropriate network parameters that characterize the particular satellite link, the collected parameters need to be entered into the web interface, shown in Figure 4, of the WANem emulator in order to get the link with the same characteristics as a real satellite link but in the laboratory for IoT communication infrastructure environment. WANem is a WAN link emulator that is able to provide the real conditions of a WAN link during the development of some particular application in a LAN environment. It does not perform any routing processes, but it possesses the ability to affect and change network parameters such as bandwidth, delay, packet loss, etc., that define the characteristics of data transmission [18,19].

However, one of the main problems of the satellite link is the unreliability of the network in the form of unexpected interruptions caused by various problems such as frequent power outages. This type of network unreliability can be simulated through random disconnection tool in an emulator.

Combination of estimated satellite link parameters with inserted expected random failure probability of network components gives the network planners a precise estimation of maritime surveillance system reliability, given the time of the satellite link outage that does not seriously affect the surveillance performance. For example, from our experience in the Gulf of Guinea, outage duration of about 9 min can be tolerated without a significant impact on the data fusion process as it is shown in [32]. With the presented laboratory for IoT communication infrastructure environment, network planners can have a good insight into potential weak spots in the overall network and a priori can plan solutions to avoid them.

## 4. Realization of IoT Communication Infrastructure Environment

Prior to the deployment of the HFSWR based surveillance and corresponding communication infrastructure, the network designer should inspect the acceptable solution using the proposed laboratory for IoT communication infrastructure environment. As mentioned earlier in the paper, a satellite link is an important and critical part of the overall communication infrastructure. Satellite transmissions face problems such as large propagation delay, low bandwidth, and high BER value that negatively affect the quality of service (QoS) that has to be guaranteed [38]. Thus, the simulation of the protected satellite link is of great importance for verification and analysis of network parameters, as well as for predicting the operation methods and behavior of developed applications in certain conditions. There are several different software and hardware commercial solutions [39,40,41] available on the market that have the ability to simulate a satellite link. In order to make the proposed solution available for a broader audience, in this paper, we have focused on an open-source solution, rather than expensive commercial simulators. Accordingly, this paper presents an open-source solution for simulating different types of protected satellite links.

### 4.1. System Architecture and Configuration

**Figure 5:**
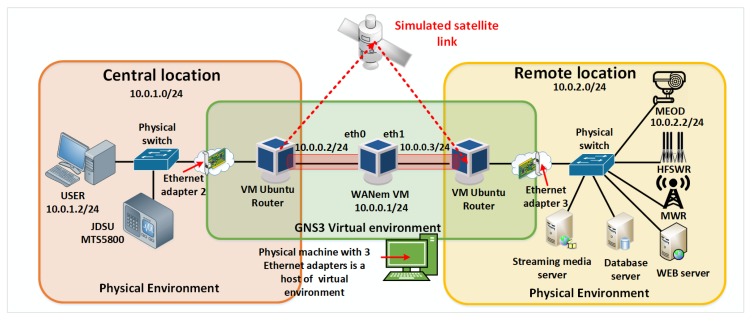
Laboratory for internet of things (IoT) communication infrastructure environment.

The proposed laboratory for IoT communication infrastructure environment is a combination of virtual and physical networks as shown in Figure 5. The reason to combine the virtual and physical networks is to increase the environment’s accuracy by reducing the number of simulated hardware devices in the proposed laboratory environment. Only the satellite link is simulated as shown in Figure 5. Using this approach, it is easy to test developed applications for HFSWR based surveillance where the satellite link simulation provides even more flexibility than it would be the case where the actual satellite link would be used. Using the satellite link simulation, the network designer can inspect and test new applications for various conditions and parameters of the satellite link, giving a great insight into the application behavior in the overall network. This also provides great flexibility to the proposed laboratory for IoT communication infrastructure environment, since it can be used during the design of maritime surveillance networks, especially those based on HFSWRs, in any other location in the world. Given that the proposed laboratory environment is open-source and economic, combined with its great flexibility, the proposed environment represents an attractive solution for surveillance and communication network design alike.

The laboratory for IoT communication infrastructure environment consists exclusively of open source, easily accessible solutions and it is built and configured in the GNS3 network simulator. GNS3 is a network simulator that allows emulation, configuration, testing and analysis of various IP based networks, as well as solving problems that occur in them. The main advantage of GNS3 is the ability to combine virtual and physical networks. However, GNS3 is not intended to replace the real router but to enable learning and testing different types of networks in a laboratory environment [20].

WANem is a software that runs on Knoppix, a Linux distribution based on Debian and it starts from VirtualBox platform within GNS3 simulator. In the proposed solution, a permanent virtual machine configured in bridge, transparent mode is created. As shown in Figure 5, WANem is primarily used to simulate satellite link in the proposed laboratory for IoT communication infrastructure environment.

The main goal of implementation of the proposed laboratory environment, shown in Figure 5, is to provide the possibility to simulate a protected IP communication channel over satellite link. The initial idea for realization of the proposed solution is to test and predict data transmission parameters of a particular system or application in development in real conditions, using different network protocols, before the system or application starts with the production.

As already mentioned, the laboratory for IoT communication infrastructure environment is realized as a combination of physical and virtual environments. The virtual environment, shown in Figure 5 in the central rectangle, is built on a physical machine that contains three Ethernet cards. It consists of GNS3 simulator with three different virtual machines, one for simulating a satellite link, and two Ubuntu machines that simulate routers at remote locations. The network interfaces of WANem virtual machine, eth0 and eth1, are configured in the bridge mode which makes the impression that routers at remote locations are connected by a point-to-point link. Bridge interface br0 has IP address 10.0.0.1/24 through which WANem web application can be accessed. The Apache http server version 2.4.13 is used to serve the WANem pages.

The described virtual environment is connected through two physical Ethernet ports with two physical switches that simulate switches at the central and remote locations. The physical switch that simulates the switch at the central location is connected to the host workstation and the JDSU MTS5800 network analyzer. On the other side, the physical switch that simulates the switch at a remote location is connected to a web server, a database server, a multimedia streaming server, and an IP camera, that are all used in the real HFSWR based surveillance system. Figure 5 shows a simplified view of the laboratory for IoT communication infrastructure environment. Some devices like firewalls are omitted for simplicity’s sake. In a similar fashion, other devices (i.e., applications) can be added to the environment. The implemented environment is adaptable to different conditions and requirements, and allows testing of some other network protocols, especially those responsible for routing and network security, as well as the behavior of some other devices that communicate via the Internet protocol over the satellite link.

### 4.2. Analyses of Eavesdropping in Satellite Link in the Proposed Laboratory

**Figure 6:**
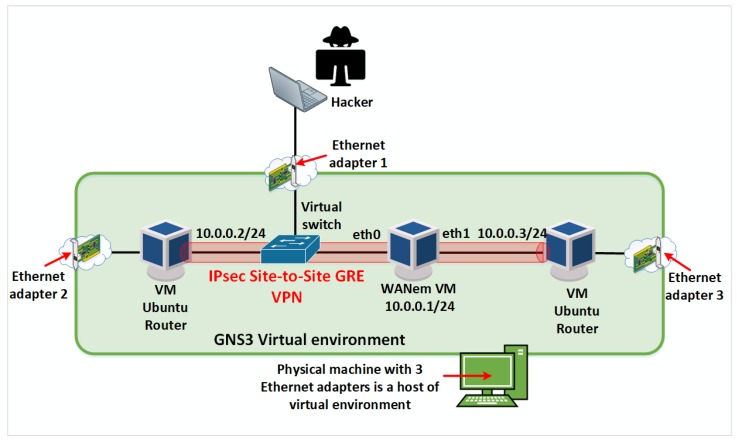
Traffic sniffing.

Since the satellite link, in the HFSWR based surveillance system would carry sensitive information, it is important to create a secured communication channel over the satellite link. Thus, the proposed laboratory for IoT communication infrastructure environment needs to enable an analysis of security risks in the network, especially over the satellite link. For the purpose of analyzing the success of establishing a secure satellite link, one virtual switch is connected between remote sites as shown in Figure 6. Analysis of packets that pass through the satellite communication channel is possible with a laptop that is connected to the virtual switch via Ethernet 1 adapter. A port mirroring is configured on the port mapped to the mentioned Ethernet card, so that all the traffic that passes through the satellite communication channel is replicated on that port. In this way, we simulate the potential attacker’s access to data carried over the satellite link.

**Figure 7:**
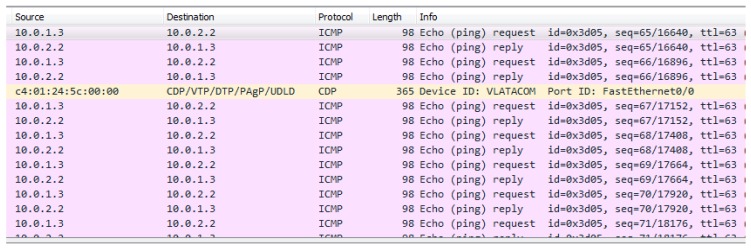
Eavesdropping detection with Wireshark (unencrypted traffic example).

In case when the satellite link protection is not configured, a hacker can eavesdrop complete traffic between remote locations using some network protocol analyzer such as Wireshark, as shown in Figure 7 [23].

In accordance with the daily challenges associated with the information security that the world is facing, the IPsec Generic Routing Encapsulation (GRE) Site-to-Site Virtual Private Network (VPN) is configured, which means that the complete IP traffic between the remote locations in the laboratory for IoT communication infrastructure environment is encrypted.

First, a GRE virtual tunnel is configured between the Ubuntu virtual routers with Quagga Routing Suite [22]. GRE is a communication protocol used to establish a direct point-to-point connection between the network nodes. It represents a simple and efficient way to transfer data through public networks such as the Internet, enabling the packet to travel through physical routers without interacting with them. GRE protocol creates the impression that routers at remote locations are connected directly by a point-to-point link. At the final destination, processes such as decapsulation and extraction of useful content (payload) are performed. On Ubuntu virtual routers, static routes to remote locations are implemented, and the packets are routed through a configured virtual tunnel.

**Figure 8:**
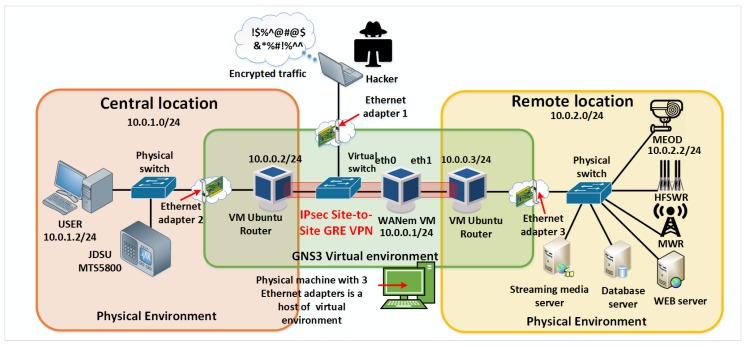
Site-to-site IPsec Virtual Private Network (VPN).

In order to make the communication channel more secure on Ubuntu virtual routers using the open-source Strong Swan platform [21], Internet Protocol Security (IPsec) [42] is configured to provide authentication and encryption of IP packets as it is shown in Figure 8.

The encryption algorithm used in this paper is the Advanced Encryption Standard (AES) 256 [43]. 

**Figure 9:**
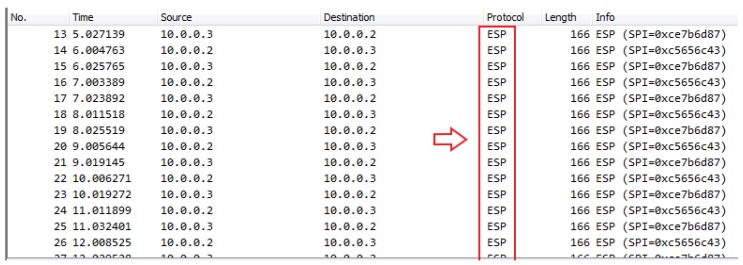
Encrypted payloads.

Once encryption is applied, the hacker is no longer able to see the contents of the packet as it is shown in Figure 9 [23].

However, configuring a protected link requires additional processing at the end of the tunnel points, which leads to degradation of system performance in regards to achieved throughput. This problem is typically solved by using some appliance that pre-accelerates VPN and provides QoS. For example, Secure Sockets Level VPN (SSL-VPN) encrypts the data but, unlike IPsec VPN, leaves the TCP headers unchanged. This means the TCP acceleration built into the satellite modem can accelerate the VPN and therefore it is considered as a simpler and more acceptable solution [38,44,45].

In this paper a typical Site-to-Site IPsec VPN solution to achieve secured communication over the satellite link is presented, however, the proposed laboratory for IoT communication infrastructure environment enables the network designer to test other security solutions as well and to analyze their impact on overall network performance.

## 5. Use cases of the Proposed Laboratory for IoT Communication Infrastructure

For multi-sensor systems, such as the one relying on the described IoT communication network, the real-time service to the end-user should not be interrupted in spite of the network instability, so multi-sensor integration algorithms must be able to compensate target data loss with predictions of target positions.

In order to maintain tracks despite the network instability, two parameters are of the utmost importance:

- Packet error rate (PER) and
- Distribution that describes the duration of network outage

The PER parameter causes loss of some target data during data transmission, while the network outage parameter causes loss of all target data.

Based on the conclusions presented above, algorithms needed for multi-sensor integration in the Gulf of Guinea are designed to compensate for sporadic data losses and for the complete absence of data. In sections that follow, we will illustrate this for two use cases that are used for systems development for the Gulf of Guinea: HFSWR data fusion and remote distance to target measurement using LRF. Finally, the third use case represents an example that shows how our proposed laboratory for IoT communication infrastructure environment can be extended to applications other than maritime surveillance applications. The first two use cases are verified in practice on a real maritime surveillance network in the Gulf of Guinea. Note that the proposed laboratory environment was extensively used during the design of the algorithms and functions given in use cases 1 and 2.

### 5.1. Use Case 1: HFSWR Data Fusion

The first example use case is a HFSWR data fusion which requires the only machine-to-machine communication with a relatively small amount of data, but communication should be in real-time. Here, itis necessary to mention that the multi-sensor data integration algorithms should not maintain tracks indefinitely long due to the input data absence. For how long the tracks should be maintained in case of input data absence is a question of both network parameters and tracked object maneuverability. Since the tracked objects (vessels of interest) at the open sea are tankers and cargo vessels, it is clear that their maneuverability is quite limited. Practically, it may be assumed that those types of vessels are generally sailing in a straight line with constant speed. Given this, the most important parameter is the duration of a network outage, as a defining factor.

**Figure 10:**
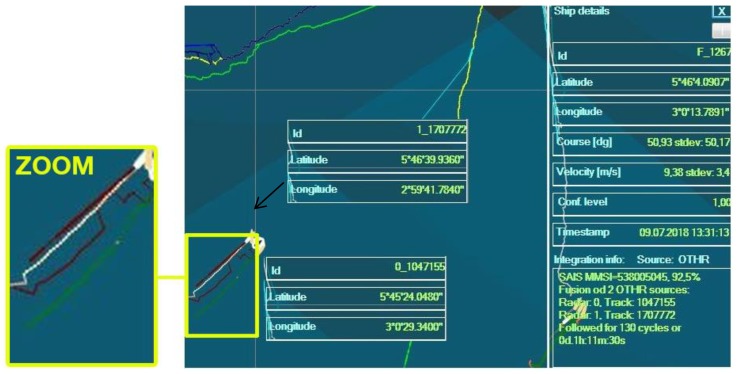
Example of multi-sensor integration (green trace: radar 0 data, red trace: radar 1 data, brown trace: fused track, white trace: Automatic Identification System (AIS) data).

Figure 10 shows a screenshot of outputs from the multi-sensor data integration algorithms before, during and after data absence for one real network situation [32].

From Figure 10, one can see that the fused track id No. F_1267 is formed from radar 0 track id No. 0_1047155 and radar 1 track id No. 1_1707772. This example also demonstrates a multi-sensor data integration before, during and after data outage, since the fused track is maintained in spite of radar 0′s erratic data availability. It can be seen that the radar 0 stopped providing target data for some time, as highlighted by the zoomed-in rectangle. During that period, the fused track was maintained entirely by the data available from the radar 1; hence, the fused track shifted toward the radar 1 track. Upon the reappearance of the radar 0 data, both radar tracks were reintegrated into a single track. This decision is justified since there is only one AIS target in the vicinity (MMSI 538005045). In this way, multiple false (duplicated) targets are eliminated. It is important to note that radar 0 track (green trace) was not broken although there was no data from the sensor (radar 0). During this period the radar 0 track was maintained by predictions.

The example presented above demonstrates a real-world scenario that took place on the 9th of July 2018, while similar scenarios are happening nearly on a daily basis.

**Figure 11:**
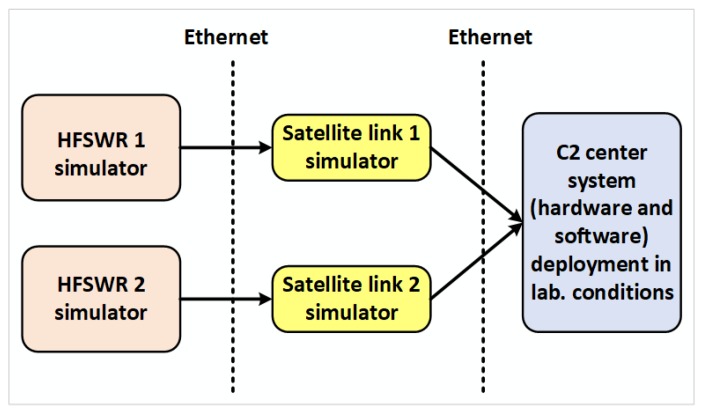
Laboratory setup for use case: the High-Frequency Surface Wave Radars (HFSWR) data fusion.

In order to obtain optimal parameters and provide uninterrupted service to the end-user, the multi-sensor data integration algorithms are firstly tested in the laboratory for IoT communication infrastructure environment with the simulated data fed through the communication network emulated by the proposed laboratory environment presented in this paper as illustrated in Figure 11. The results of these tests can be found in [31,32].

### 5.2. Use Case 2: Remote Distance to Target Measurement Using LRF

The second use case is a scenario of MEOD usage: remote distance to target measurement using LRF. It is more complex compared to the first one, because it combines live video streaming, satellite link and high level of interactivity with human operators. In this use case, there are a lot of trade-offs and challenges like:

- Live video streaming requires high bandwidth, which might be very expensive when using satellite link as transmission media
- Operator user experience requires the highest possible resolution and frame rate of video stream
- Targeting for distance measurement is performed by pan-tilt positioner, whose control should be smooth for a good user experience
- LRF can perform very limited measurement within a time frame (e.g., six measurements per minute) due to its construction, because the laser needs to be re-charged after measurement
- Usage of satellite link gives a huge delay which makes measuring triggering difficult for moving target

**Figure 12:**
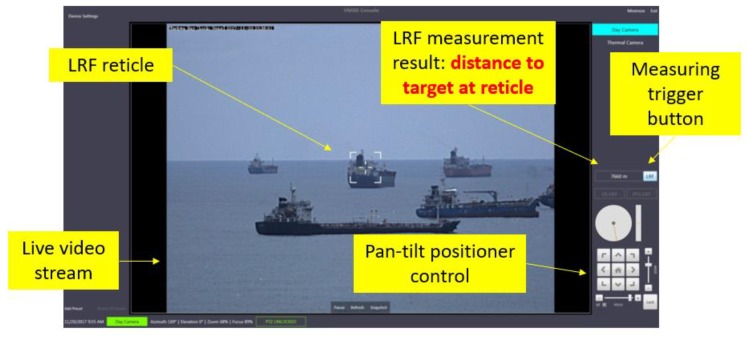
Laser Range Finders (LRF) use case: user interface for remote distance to target measurement.

Thus, the design of proper user interface and video streaming parameters is very crucial. In Figure 12 the final solution of the user interface is shown. The operator monitors the control video stream and uses pan-tilt positioner control to put LRF reticle on the target. Measurement is initiated by pressing the measurement trigger button (please note that the button is close to the positioner control so it could be easily clicked by a mouse). The measurement result is shown in a measurement result window. The image shown in Figure 12 is taken on the Gulf of Guinea and the distance between MEOD and ship target, in this case, was 7.6 km. The measurement is performed from C2 center which was 500 km away from MEOD via satellite link.

**Figure 13:**
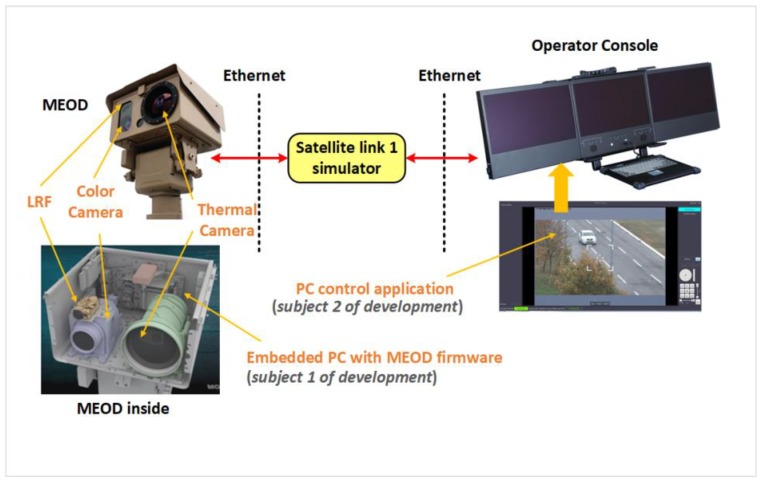
Laboratory setup for use case 2: distance to target measurement using LRF.

The development of the MEOD operator console software and MEOD firmware was done in Belgrade, Serbia, far away from the installed system. For a real simulation of described real scenario usage, the laboratory setup shown in Figure 12 was used. Note that only parts of the laboratory for IoT communication infrastructure environment (shown in Figure 5) relevant for this use case are shown in Figure 13. MEOD is connected to the operator console via a satellite link simulator. Instead of naval scene with measuring the distance to the ship, the scene with slowly moving cars is used. The satellite link simulator is used for development PC control application and MEOD firmware with optimization of image compression parameters. The final outcome of this development is Motion JPEG (MJPEG) image streaming with 7 frames to second at a resolution of 1280 × 720 pixels, which gives optimal trade-off regarding user experience and occupied satellite link bandwidth. Additionally, a fast application recovery from a high PER occurrence or temporary link down-state is successfully developed.

The described use case is acceptable for slow targets like ships that are in a laboratory scenario simulated by slowly moving vehicles. This is not acceptable for fast targets like airplanes or drones, so the additional target detection and target tracking by pan-tilt positioner movement should be developed. For this development, the proposed laboratory for IoT communication infrastructure environment is a very useful tool that helped significantly in speeding up the development process.

### 5.3. Potential Expansions of the Proposed Laboratory for IoT Communication Infrastructure Environment

**Figure 14:**
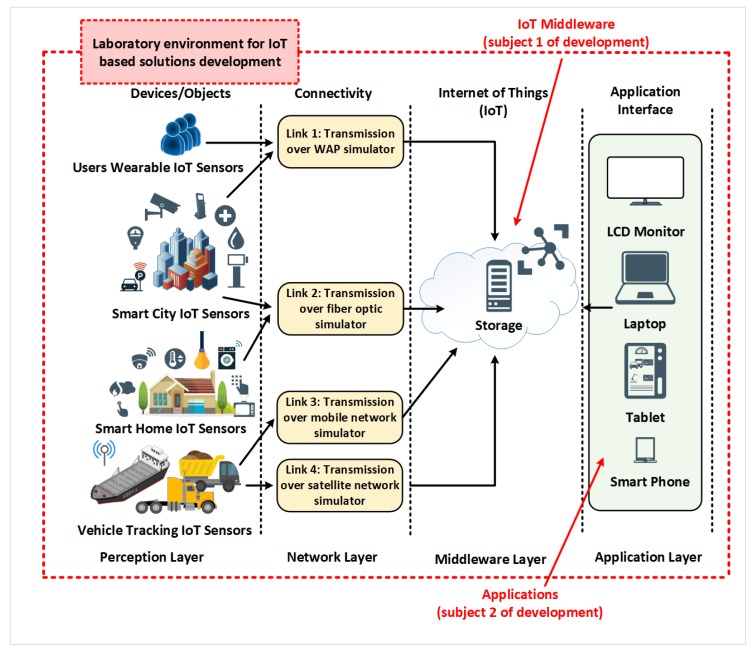
Application in IoT solutions development laboratory.

Since the described laboratory for IoT communication infrastructure environment is designed by using open source components, it is a very cost-effective solution and could be used for IoT laboratory environment for Smart City applications. The common IoT ecosystem and IoT laboratory are described in [46]. The majority of IoT solutions development is related to the development of middleware and application layers, while the device layer is usually taken as is by section of proper devices from the IoT market. Network layer transmission effects strongly influence the successful design of IoT based applications. The proposed laboratory environment, shown in Figure 5, can also be configured to simulate link types other than satellite links, like a wireless access point, optical links, and others as shown in Figure 14. The use case 3 shows the versatility of our proposed laboratory for IoT communication infrastructure environment.

## 6. Conclusions and Future Work

In this paper, a laboratory for IoT communication infrastructure environment for remote maritime surveillance in the Equatorial areas is presented. The presented laboratory environment is designed in order to facilitate maritime sensor network design process in areas where the communication network is dependent on data transfer over the satellite links. Since this is a common case in the Equatorial areas, the real data used during the laboratory for IoT communication infrastructure environment development are collected during maritime surveillance network development in the Gulf of Guinea. Successful development of the presented laboratory environment greatly facilitates the development of critical maritime surveillance network functions, such as data integration algorithms, especially those used for multi-sensor integration at over the horizon distances. The main advantage of the proposed laboratory environment is the inclusion of satellite link simulation which significantly extends the scope of the supported IoT solutions when compared to other existing IoT laboratories. The usage and performances of the proposed laboratory environment are verified by the development of the real maritime surveillance fully operative network in the Gulf of Guinea.

The proposed method of simulating network effects can be used to develop and test not only maritime surveillance applications, but the same concept can be used to simulate others surveillance environments such as land border surveillance (where additional needed sensors for example “smart fence”) or smart city surveillance (where automatic number plate recognition (ANPR) sensors are needed). The proposed method could also be applied in surveillance networks based on sensors mounted on flying platforms, where packet loss parameters should be described with more complex channel models, which is a task for our future research.

## Figures and Tables

**Table 1:** Real sensor network requirements.

Sensor Type	HFSWR	MEOD	MWR
Protocol	TCP	UDP	TCP
Traffic type	Periodic	Continuous	Continuous
Throughput	64 Kbps	512 Kbps	64 Kbps
Latency	Less than 3 s	Less than 1 s	Less than 2 s
Jitter	Less than 0.5 s	Less than 0.1 s	Less than 0.3 s

**Table 2:** Iperf3 measurements of satellite link parameters.

Measurement ID	Generated Traffic	Throughput	Jitter	Packet Loss	Protocol
Measurement 1	2.5 Mbit/s	2.37 Mbit/s	6.0 ms	5.1%	UDP
Measurement 2	2.5 Mbit/s	2.47 Mbit/s	7.4 ms	1.1%	UDP
Measurement 3	2.5 Mbit/s	2.47 Mbit/s	4.6 ms	1.1%	UDP
Measurement 4	2.5 Mbit/s	2.48 Mbit/s	5.9 ms	0.71%	UDP
Measurement 5	2.5 Mbit/s	2.48 Mbit/s	7.4 ms	0.71%	UDP
Measurement 6	N/A	0.18 Mbit/s	N/A	N/A	TCP (1)
Measurement 7	N/A	1.9 Mbit/s	N/A	N/A	TCP (10)
Measurement 8	N/A	2.63 Mbit/s	N/A	N/A	TCP (15)
